# Gallbladder in the Wrong Neighbourhood: Tackling Para-Urostomal Herniated Cholecystitis With Open Cholecystostomy

**DOI:** 10.7759/cureus.79543

**Published:** 2025-02-24

**Authors:** Supun M Bakmiwewa, Ashish Vaska, Marwan Idrees, Yasser Farooque

**Affiliations:** 1 Department of Upper Gastrointestinal Surgery, Liverpool Hospital, Sydney, AUS; 2 Faculty of Medicine, University of New South Wales, Sydney, AUS

**Keywords:** acute cholecystitis, co-morbidities, elderly, hernia, percutaneous cholecystostomy

## Abstract

Acute cholecystitis is usually managed with a cholecystectomy. Percutaneous cholecystostomy serves as a viable alternative when cholecystectomy is not appropriate. While parastomal hernias are a frequent complication of stomas, we present an unusual case of an elderly man with acute cholecystitis developing within a para-urostomal hernia. Given his significant comorbidities and personal preference, an open/surgical cholecystostomy under local anaesthesia was successfully performed, demonstrating a tailored approach to the management of a complex clinical scenario.

## Introduction

Acute cholecystitis is an acute infection and inflammation of the gallbladder, commonly due to cholelithiasis [1]. Cholecystectomy is generally the standard management for acute calculous cholecystitis depending on patient and disease factors [2]. Percutaneous cholecystostomy is an alternative management option and is utilised in patients who cannot safely undergo a surgical procedure [2,3]. It involves imaging-guided drain insertion to the gallbladder for decompression and treatment of acute infection. Open cholecystostomy is another alternative, although uncommon due to the technically challenging sub-hepatic location of the gallbladder [4].

Parastomal hernias involve the herniation of abdominal contents through a defect in the abdominal wall surrounding an existing stoma [5]. Management depends on the hernia contents and the presence of obstruction or strangulation and ranges from non-operative management to urgent surgical intervention for compromised bowel [6].

This case report describes the management of acute calculous cholecystitis within a para-urostomal hernia by performing an open/surgical cholecystostomy under local anaesthesia.

## Case presentation

An 88-year-old male presented with a four-day history of painful para-urostomal swelling and associated anorexia, vomiting, pain, and fever. However, his urostomy remained active. His past medical and surgical history was extensive and included previous radical cystoprostatectomy and ileal conduit for prostate and bladder cancer, metastatic melanoma on palliative immunotherapy, Parkinson's disease with severe oropharyngeal dysphagia, atrial fibrillation on warfarin, and known cholelithiasis. Despite this, the patient was cognitively intact and lived at home with minimal support.

**Figure 1:**
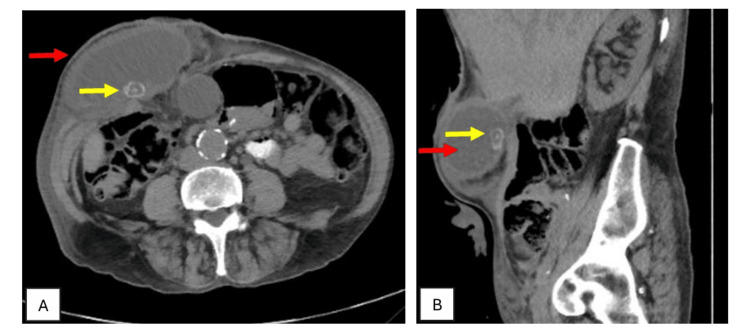
Computed tomography scan of the abdomen and pelvis showing parastomal hernia containing the gallbladder (red arrow) with cholelithiasis (yellow arrow) and cholecystitis. (A) Transverse section. (B) Sagittal section.

On review in the district hospital emergency department, he was haemodynamically normal, afebrile but tachypneic. On examination, he had a large irreducible and tender parastomal hernia with some inflammatory changes in the overlying skin but a healthy and active urostomy. Admission blood results were as follows: white blood cell count of 14.3 x10^9^/L, C-reactive protein at 324 mg/L, haemoglobin (Hb) at 78 g/L, and an acute kidney injury with creatinine >200 µmol/L. He underwent computed tomography (CT) of the abdomen and pelvis with no contrast initially, which demonstrated a parastomal hernia containing a distended gallbladder with gallstones and evidence of cholecystitis (Figure 1) and associated obstruction of the ileal conduit (likely due to the gallbladder), with bilateral hydroureteronephrosis. He was reviewed by the local general surgical team, commenced on intravenous antibiotics, and transferred to our tertiary hospital under the upper gastrointestinal/hepatobiliary unit.

Extensive discussions were done with the patient and his family regarding the best management course given his significant co-morbidities. The patient and his family had a clear understanding of the situation and declined significant operative intervention, requesting the least invasive treatment to manage the clinical situation. Thus, an open surgical cholecystostomy under local anaesthesia rather than parastomal hernia repair and cholecystectomy was offered.

**Figure 2:**
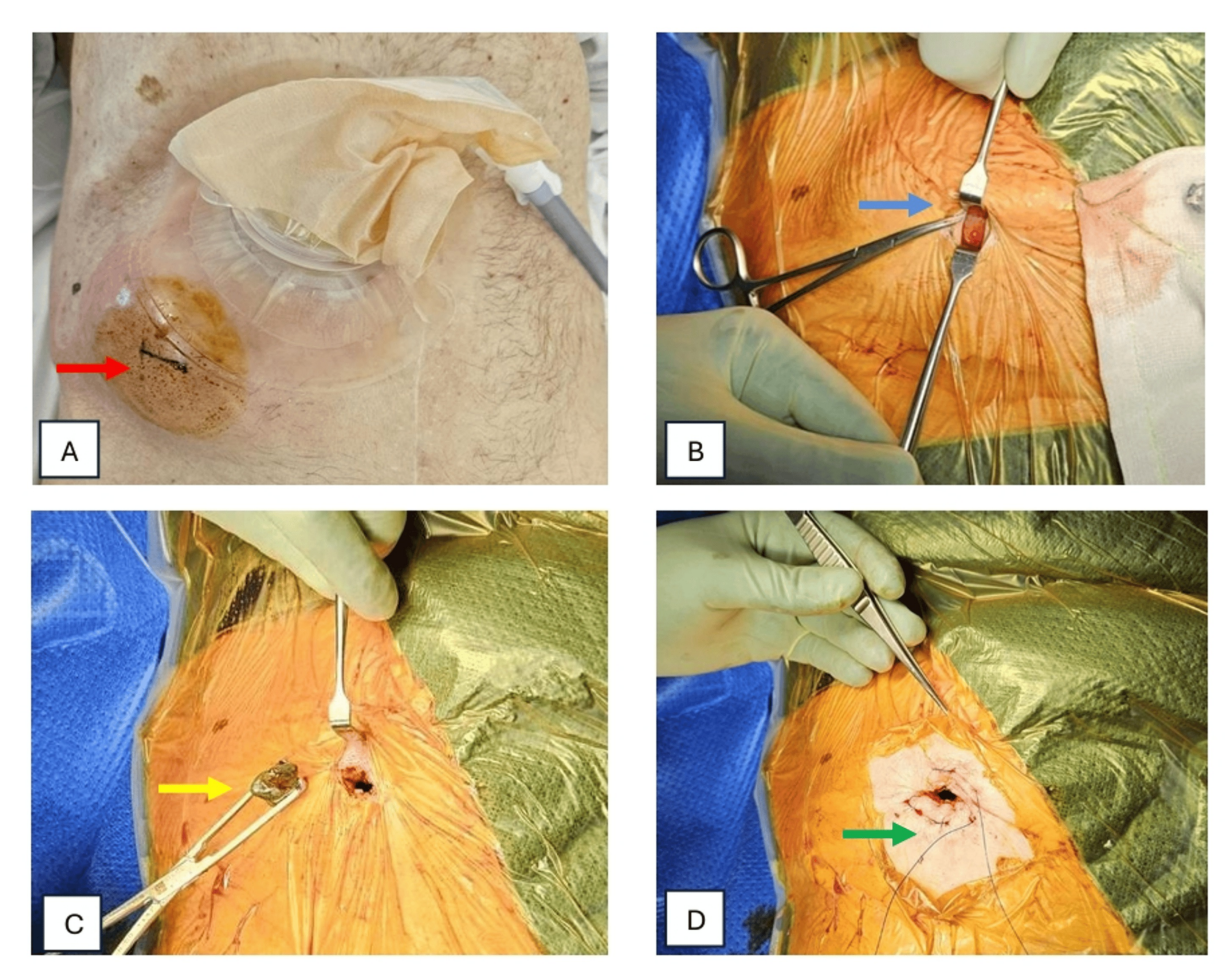
Open surgical cholecystostomy under local anaesthesia. (A) Site marked and prepared with Betadine (red arrow). (B) The gallbladder was identified after incising the hernia sac (blue arrow). (C) The gallbladder was incised and stones were retrieved (yellow arrow). (D) The gallbladder was marsupialised and a cutaneous purse string suture was applied (green arrow).

**Figure 3:**
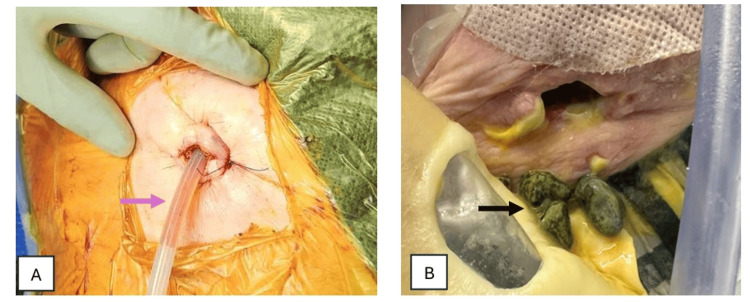
Open surgical cholecystostomy under local anaesthesia. (A) A 20-Fr three-way Foley catheter (purple arrow) was used as a cholecystostomy tube and secured with a prolene purse string suture. (B) The cholecystostomy tube was removed (day 28 from surgery) and further stones were extracted (black arrow).

He underwent open cholecystostomy via the parastomal hernia with a drain insertion under local anaesthesia on day two of admission. Local anaesthesia with 1% lignocaine and 0.75% ropivacaine with adrenaline was infiltrated surrounding the planned surgical site, followed by site preparation with Betadine (Figure 2A). A 2-cm incision was made at the right lower quadrant at the level of the umbilicus corresponding to the imaging findings. The gallbladder was identified after incising the hernia sac (Figure 2B), which was then incised and stones were retrieved (Figure 2C). Next, the gallbladder was marsupialised and a cutaneous purse string suture (2-0 prolene) was applied (Figure 2D). A 20-Fr three-way Foley catheter was used as a cholecystostomy tube and was secured with the prolene purse string and 15 mL of water placed into the balloon (Figure 3A). The drain was flushed with 10 ml of normal saline twice daily. He was treated with antibiotics based on the findings from the intra-operative samples (bile sample positive for *Serratia marcescens* and *Klebsiella oxytoca*) and recovered well postoperatively. The patient was discharged on postoperative day seven with a plan for further oral antibiotics, community nursing for ongoing daily normal saline drain flushes, and specialist follow-up.

The patient re-presented to the hospital on postoperative day 26 with concerns regarding the surgical wound. The open cholecystostomy drain site had a pressure necrosis injury suspected to be a result of inadvertent overinflation of the Foley catheter balloon during drain flushes. This was managed conservatively with wound care and a short course of intravenous antibiotics. The cholecystostomy drain was removed on day two of readmission as a tract had been formed and a wound drainage bag was applied to the site. His open cholecystostomy tract remained patent and further gallstones were extracted during the admission (Figure 3B). The patient was discharged on day four of readmission. He remained asymptomatic from his gallstone disease from discharge until his death from an unrelated medical cause eight months later.

## Discussion

Surgical management of acute cholecystitis has its own complications as with any other procedure. These include general risks, including anaesthetic-related risks, skin infection, pain, venous thromboembolism and specific risks of damage to surrounding structures (common bile duct, duodenum, liver), bleeding, bile leak, and retained stones [7]. Whether a cholecystectomy is offered to a patient is determined by patient, disease, and clinical factors, including fitness for surgery [2].

Parastomal hernias are a common complication of stomas and urostomal hernias are known to be more symptomatic than hernias associated with ileostomy/colostomy [5]. Surgical repair of the hernia may be indicated if stoma function is impaired or if the hernia contents become compromised. Surgical repair of urostomal hernias can be technically challenging with a high risk of recurrence and a risk of ureteric injury [8,9]. These risks need to be balanced against the potential benefits of intervention for the patient.

Gallbladder-containing parastomal hernias are rare and para-urostomal herniations even more so. Olusola et al., in their systematic review of parastomal gallbladder herniations, identified 18 case reports, including three publications with literature reviews [6,10-12]. Our search across PubMed, Embase, and Google Scholar with the keywords “cholecystitis” and “parastomal hernia” for the last 20 years, augmented by snowballing, returned 17 unique case reports, including one case that had been published twice and included as two separate cases in Olusola et al.'s study. There were four case reports of para-urostomal herniation in the literature (Table 1). The first case of a para-urostomal hernia containing the gallbladder was in 2005 of a female with a right lower quadrant ileal conduit who had presented to the hospital with an acute incarceration of a parastomal hernia containing the gallbladder [13]. She underwent a cholecystectomy via a parastomal hernia approach with simultaneous hernia repair. The latest case was in 2024 and described a 90-year-old male with acalculous cholecystitis with the gallbladder in an urostomal hernia where he underwent open cholecystectomy [14]. The majority (10/17) of published cases with cholecystitis within a parastomal hernia underwent simultaneous cholecystectomy and hernia repair.

**Table 1:** Summary of previously published cases of para-urostomal hernia containing the gallbladder. GA: general anaesthesia; LUQ: left upper quadrant; M: male; F: female; Y: yes; N: no.

Publication	Age/gender	Cholecystitis	Type of hernia	Intervention	Surgery details	Hernia repair (Y/N)
Shearer et al. (2024) [14]	90 M	Y, acalculous	Para-urostomal	Failed medical management leading to surgery	GA, upper paramedian and parastomal incisions, top-down cholecystectomy	N
Seang et al. (2022) [15]	87 F	Y, perforated	Para-urostomal	Surgery	GA, diagnostic laparoscopy via LUQ cutdown, upper midline open cholecystectomy	N
To et al. (2015) [16]	85 F	Y	Para-urostomal	Surgery	GA, midline laparotomy, cholecystectomy	N
St Peter et al. (2005) [13]	73 F	Y	Para-urostomal	Surgery	GA, open cholecystectomy via parastomal incision	Y, primary

In this case, an open cholecystostomy under local anaesthesia rather than cholecystectomy and hernia repair was performed due to patient clinical factors and preference. Operative access was straightforward due to the migration of the gallbladder into the parastomal hernia sac. We chose an open cholecystostomy approach due to the anatomical variation rendering this approach a simple procedure with a better drainage capability as well as the capacity to simultaneously retrieve/clear gallstones as compared to percutaneous cholecystostomy. We utilised a 20-Fr three-way Foley catheter to have a larger calibre drainage tube and to also allow for simultaneous drainage/washout if needed later (a three-way catheter was used due to a shortage of two-way catheters larger than 18 Fr in the hospital supply). This procedure met clinical objectives to control sepsis and manage symptoms and satisfied the patient’s treatment goals for limited surgical intervention.

## Conclusions

Acute calculous cholecystitis within a para-urostomal hernia is a rare clinical entity and managing this condition in a patient with complex comorbidities presents a significant challenge. This case report describes open/surgical cholecystostomy under local anaesthesia as a management option for acute calculous cholecystitis within a gallbladder containing para-urostomal hernia, adding a further operative option to the surgeon’s armamentarium for this unusual pathology. This is the first recorded case in the English literature describing this management approach.
